# One More Central Authority for Charity

**Published:** 1908-01-18

**Authors:** 


### Editor's Letter-Box.

### ONE MORE CENTRAL AUTHORITY FOR CHARITY.

"Hospital Secretary" writes :—We are threatened by another central authority, this time on a tremendous scale, to control not only the hospitals but every other charity and, indeed, every philanthropic movement, including the great friendly societies and benefit clubs, with a side-issue that will not leave untouched the Poor-law and parochial grants for the unemployed. For some time there have appeared in the *Times* long articles condemnatory of the management of every form of charitable relief, till at last the perusal of these articles makes one ashamed of being associated with an institution for the care of suffering humanity. These articles have been contributed by "a correspondent" whose identity is as firmly established by a perplexity of verbosity as if it had been written on the note-paper of the Athenæum Club. Furthermore, these articles are now backed up by a leader in the same paper that bears the impress of being written by the author of the articles.

That there is a large amount of fact and good logic no one will deny, and one feels that it is very kind of the writer to assert that "there is no indictment of the administration of certain charities." We are told that there are three organisations in London striving to bring about the co-operation of charities. They are (1) the Charity Organisation Society; (2) the King Edward's Hospital Fund aided by the Hospital Saturday Fund and the Hospital Sunday Fund; and (3) the City of London Council for Organisation of Charity. This is rough on the Central Hospital Council; but now the position is that there shall be a great council of organisation that is to knit together and advise every other organisation, no matter what form the charity may take, with a view to the avoidance of overlapping and the shutting out of those who do not deserve help or who are getting too much. The idea is a splendid one, and it is large. There is room enough here in all conscience for organisation; if it ever comes into being let us hope it will leave some room for charity also.

The King's Fund is commended by the writer, and with justice, and he acknowledges that the success that has been attained by its efforts is due to the power of the purse; he does not seem so ready to acknowledge that the importance of the Charity Organisation Society is the lack of that stimulus. Instead of the proposal for a huge central organising body without any power to enforce its dictates, would it not be well to follow the lead of the successful King's Hospital Fund and establish a King's Convalescent Fund, a King's Cripples' Fund, a King's Orphanage Fund, etc.? When they are in as good a position to control these groups, there will be time enough to consider a working arrangement between the various funds with some prospect of success.

The public is likely to be misled by such articles as these, no matter how good the intention. To talk about such measures being necessary to attain "sound, practical, and economic" methods, is to give the idea to the uninitiated that things are in a very bad way indeed in the charity world. The last twenty-five years' work has shown this not to be the case; in the hospitals, at any rate, a larger amount of good work is being done every year at a lower cost. May we be defended for a while from another organising body!

